# Nationwide trends in molecular epidemiology of methicillin-resistant *Staphylococcus aureus*, Finland, 1997–2004

**DOI:** 10.1186/1471-2334-7-94

**Published:** 2007-08-14

**Authors:** Anne-Marie Kerttula, Outi Lyytikäinen, Minna Kardén-Lilja, Salha Ibrahem, Saara Salmenlinna, Anni Virolainen, Jaana Vuopio-Varkila

**Affiliations:** 1 Department of Microbiology, National Public Health Institute, Helsinki, Finland; 2 Department of Infectious Diseases Epidemiology, National Public Health Institute, Helsinki, Finland; 3 Department of Bacteriology and Immunology, Haartman Institute, Helsinki, Finland; 4 Department of Clinical Microbiology, Turku University Hospital, Turku, Finland

## Abstract

**Background:**

In Finland, the annual number of MRSA notifications to the National Infectious Disease Register (NIDR) has constantly increased since 1995, and molecular typing has revealed numerous outbreak isolates of MRSA. We analyzed the data on MRSA notifications of the NIDR, and MRSA isolates were identified mainly by pulsed-field gel electrophoresis (PFGE) at the National Reference Laboratory (NRL) in Finland during 1997–2004. One isolate representative of each major PFGE type was further characterized by multilocus sequence (MLST)-, staphylococcal cassette chromosome *mec *(SCC*mec*)-, and Panton-Valentine leukocidin (PVL)-typing.

**Results:**

The annual number of MRSA notifications to the NIDR rose over ten-fold, from 120 in 1997 to 1458 in 2004, and the proportion of MRSA among *S. aureus *blood isolates tripled, from <1% during 1997–2003 to 2.8% in 2004. During the same period of time, 253 different strains among 4091 MRSA isolates were identified by PFGE: 215 were sporadic and 38 outbreak/epidemic strains, including 24 new strains. Two epidemic strains resembling internationally recognized MRSA clones accounted for most of the increase: FIN-16 (ST125:IA) from <1% in 1997 to 25% in 2004, and FIN-21 (ST228:I) from 6% in 2002 to 28% in 2004. Half of the ten most common strains carried SCC*mec *IV or V.

**Conclusion:**

The predominant MRSA strains seem to change over time, which encourages us to continue implementing active control measures with each new MRSA case.

## Background

Methicillin-resistant *Staphylococcus aureus *(MRSA) has become a persistent problem worldwide. MRSA has been established as a major hospital pathogen but it has also been found increasingly in long-term facilities, and in communities from persons having no connection to the health-care setting [1]. It is generally accepted that hospital-acquired MRSA isolates spread to the long-term facilities as well as to the community. However, the community-based isolates may migrate into the health-care settings, and thus create a two-way flow of MRSA [2].

Of the genotyping methods, pulsed-field gel electrophoresis (PFGE) has been suggested to be the golden standard for local outbreak investigations [3], and multi locus sequence typing (MLST) and the mobile genetic element, staphylococcal cassette chromosome *mec *(SCC*mec*)-typing [4,5] for understanding the evolution of different MRSA clones [6]. SCC*mec *types I-III, and IVA have been associated with hospital acquisition, and types IV and V are mostly found in strains circulating in the community [7]. Several major different pandemic MRSA clones have been identified mainly by PFGE, i.e. Iberian, Brazilian, Hungarian, New York/Japan, Pediatric, and UK EMRSA-15 and -16 clones [8]. MLST has, however, revealed that several MRSA clones, considered to be distinct by using PFGE and other molecular typing techniques, were indistinguishable [9]. Using MLST and eBURST, the five most common pandemic MRSA lineages can be grouped as clonal complexes: CC5, CC8, CC22, CC30, and CC45. Several nationwide studies on molecular epidemiology of MRSA have also been performed [10-14]. However, the extensiveness of the MRSA isolate collections used in those studies varied, and most of the studies were based on selected isolates as opposed to all MRSA isolates being identified on the national level.

The purpose of our study was to examine the molecular epidemiology of MRSA in Finland during 1997–2004 in comparison to European and pandemic MRSA clones by using the nationwide collection including all MRSA isolates, thus giving an insight into the trends of MRSA evolution at the nationwide level over a certain period of time. We also performed a survey which focused on the background information of the MRSA-positive persons in 2001–2003.

## Results and Discussion

**Figure 1:**
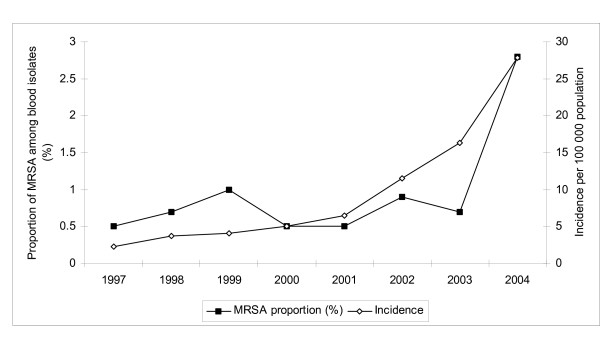
Annual incidence of MRSA notifications per 100,000 population, and the proportion of MRSA among *S. aureus *blood isolates, Finland, 1997–2004.

Our study is based on comprehensive collection of MRSA isolates obtained nationwide, and during 1997–2004, 4026 MRSA notifications were received by the NIDR (120–1458 per year). The annual incidence of MRSA rose over ten-fold, from 2.3 notifications per 100,000 population in 1997 to 27.9 in 2004 (Figure 1). Among the 4026 MRSA notifications, 71 specimens were obtained from blood and 4 from CSF; 30 of the blood isolates and 3 of the CSF isolates were from 2004. The proportion of MRSA among blood isolates of *S. aureus *rose from under 1% to 2.8% in 2004, although it is still low compared with many other countries in Europe or worldwide [15,16]. During 1997–2004, the numbers of MRSA notifications were strikingly high in three HDs and in six others there was an increasing trend. In 1997, the incidence of MRSA was slightly over 10 notifications per 100,000 population only in the Helsinki metropolitan area, whereas the incidence rate rose to over 20 notifications per 100,000 population in eight HDs by 2004.

**Figure 2:**
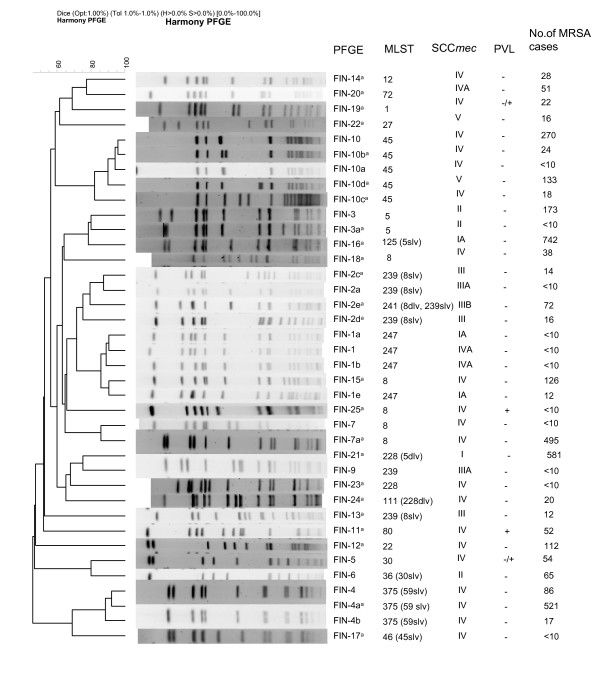
Dendrogram of the epidemic MRSA strains based on the Dice coefficient of pattern similarity, obtained after *Sma*I macrorestriction analysis of DNA of MRSA isolates. Strains which were found first time in Finland during 1997–2004 are indicated by "a". Slv means single locus variant, and dlv means double locus variant.

During the same period of time a total of 4091 MRSA isolates were studied in the reference laboratory. PFGE identified 253 different strains of which 38 were outbreak/epidemic strains, and 215 sporadic (Figure 2). Twenty-four new outbreak/epidemic strains appeared during 1997–2004. The proportion of sporadic strains varied between 3–13% during the study period. There is a slight difference in the numbers of MRSA notifications and MRSA isolates (65), this is partly due to the differences in the case definitions (a time interval of 12 months versus 36 months); also if the same person had two different types of isolates, both of them were included (a total of 27 persons carried two different strains). There may also exist some MRSA notifications of which the MRSA isolate was not sent to the reference laboratory and vice versa.

At the beginning of the study period (1997–2001), the distribution of different MRSA strains was more diverse, but since 2002, five strains (FIN-16 (ST125:IA), FIN-4 (ST375:IV), FIN-21 (ST228:I), FIN-7 (ST8:IV), and FIN-10 (ST45:IV and V) have been most predominant (Table 1). The number of internationally recognized epidemic strains, FIN-16 (ST125:IA), a single-locus variant of ST5 (New York/Japanese clone) – named previously Bel EC-3 – and FIN-21 (ST228:I), has strongly increased since 2003. In 2004 more than half of the isolates (53%) were composed of FIN-16 and FIN-21. The most common MRSA isolates found from blood and from CSF in 2004 were FIN-16 (12/34), of FIN-21 (9/34), and of FIN-4 (4/34); no significant associations were found between the type of specimen (blood and CSF vs. other) and these strains in 2004 nor during the whole study period.

**Table 1:** Ten most common Finnish epidemic MRSA strains analyzed by PFGE (one per person) 1997–2004.

^a^MRSA strain	Sequence type (ST) and SCC*mec *type	1997	1998	1999	2000	2001	2002	2003	2004	Overall	No. of hospital districts in which the strain found (total n = 20)
FIN-16	ST125:IA	1	2	6	3	21	103	231	375	742	15
FIN-4	ST375:IV	33	39	46	65	93	72	94	183	625	20
FIN-21	ST228:I	0	0	0	0	0	32	118	431	581	3
FIN-7	ST8:IV	4	12	12	10	41	145	83	188	495	16
FIN-10	ST45:IV, V	15	16	16	48	28	69	146	110	448	17
FIN-3	ST5:II	25	21	8	4	9	16	46	44	173	14
FIN-15	ST8:IV	0	0	0	25	9	27	32	33	126	7
FIN-12	ST22:IV	2	9	3	13	14	22	22	27	112	16
^b^FIN-2	ST241:IIIB	0	6	44	8	4	3	2	5	72	7
FIN-6	ST36:II	18	14	7	6	11	7	1	1	65	11
Other strains		27	59	53	35	61	58	59	85	437	20
Sporadic		12	19	17	32	33	25	31	46	215	19
Total		137	197	212	249	324	579	865	1528	4091	

^a^MRSA strains include related patterns with a 1–6 band differences.

^b^This represents only FIN-2e subtype.

The proportion of FIN-16 (ST125:IA) strains increased from <1% in 1997 to 25% in 2004. The majority of all FIN-16 strains (554/742) were found in one HD in central Finland, and it has dominated in that area since 2001, but it also extended to 14 other HDs. Pérez-Roth and others have reported a similar increase of ST125:IV strains in Spanish hospitals [17]. Our survey (2001–2003) showed that FIN-16 strains were more likely to be found from clinical specimens than from those obtained on screening and other basis (199 [25%] of 805 vs. 160 [18%] of 902; p < 0.05) (Table 2), and it was the most common strain found from invasive specimens in 2004 indicating that FIN-16 might be of epidemic potential and virulent. Since FIN-16 has dominated in one HD for four years it can be considered an endemic strain in that area, especially in long-term facilities. Thus, we can equally assume that its capacity to cause clinical infections may occur because FIN-16 strain has reached a sufficiently high level in the population, especially among the oldest and most seriously ill patients who are being transferred between long-term facilities and acute care hospitals.

**Table 2:** Background information on patients and staff members who tested positive for the ten most common epidemic MRSA strains, 2001–2003. The response rate of the survey was 97% (1707/1768).

MRSA strain	Number of patients (n = 1598)					Number of staff members (n = 109)				Total no. of isolates
		
	Sequence type (ST) and SCC*mec *type	Clinical specimen	^a^Screening specimen; exposure to MRSA	^b^Screening specimen; hospital contact abroad	Other reason for taking the sample	Clinical specimens	^c^Screening specimen; epidemic situation	^d^Screening specimen; hospital contact abroad	Other reason for taking the sample	
FIN-16	ST125:IA	197	131	5	18	2	3	1	2	359
FIN-7	ST8:IV	70	159	1	6	-	28	-	-	264
FIN-4	ST375:IV	163	56	5	12	4	8	-	-	248
FIN-10	ST45:IV, V	87	129	2	6	2	12	-	-	238
FIN-21	ST228:I	58	82	1	3	-	1	-	-	145
FIN-3	ST5:II	22	30	8	7	-	4	-	-	71
FIN-15	ST8:IV	34	19	-	5	-	9	-	1	68
FIN-12	ST22:IV	19	12	8	4	-	1	14	-	58
FIN-6	ST36:II	5	1	5	-	-	1	1	-	13
^e^FIN-2	ST241:IIIB	7	-	2	-	-	-	-	-	9
Other strains		79	49	8	2	3	5	2	2	150
Sporadic		52	22	19	2	1	1	1	-	98
Total		793	690	64	65	12	73	19	5	^f^1721

^a^Screening specimens of MRSA increased from 36% (96/265) in 2001 to 45% (364/816) in 2003.

^b^Hospitalized or undergone a surgical procedure outside the Nordic countries within 6 months

^c^Screening specimens of MRSA increased from 46% (12/26) in 2001 to 82% (36/44) in 2002, but decreased to 64% (25/39) in 2003.

^d^Worked outside the Nordic countries within 6 months

^e^This represents only FIN-2e subtype.

^f^The total number of strains (1721) exceeds the number of MRSA-positive persons (1707) by 14, because in some cases more than one reason for sampling was chosen in the questionnaire.

The FIN-21 (ST228:I) strain was found in Finland for the first time in September 2002 [18]. Its proportion increased from 6% in 2002 to 28% in 2004, and it occurred mostly in the Helsinki metropolitan area (98%; 572/581) where it caused many epidemic clusters during 2002–2004. The strain has a similar PFGE pattern to the Italian clone [12], expresses MLST allelic profile ST 228, a double-locus variant of ST5, and SCC*mec *type I. MRSA strains with the same ST228 have also been reported as the "Southern Germany" epidemic clone [19]. The strain tended to be found more often from screening and other specimens than from those taken on clinical basis (58 [7%] of 805 vs. 87 [10%] of 902; p = 0.086) (Table 2), as opposed to FIN-16. This may be due to differences in the MRSA screening activity between the HDs, or the later appearance of the strain in Finland. We have previously shown that during 2001, the screening activity varied widely between the HDs (0–2768 per 100,000 population), but it was not related to the rate of MRSA [20].

UK EMRSA-16, currently named as FIN-6 (ST36:II), decreased from 13% in 1997 to <1% in 2003, and FIN-3 (ST5:II) from 18% to 3%, respectively. UK EMRSA-15, now FIN-12 (ST22:IV) varied between 1–6%. This is in contrast to the situation in the UK where the same strains (UK EMRSA-15 and -16) are predominant [21]. In Spain a rapid nosocomial dissemination of an identical strain (UK EMRSA-16) has been reported [17], and also in Sweden it caused a large outbreak in hospital setting [22]. In our material, FIN-12 was the most commonly found strain, 14/19 (74%), among staff members screened because of hospital contact abroad (Table 2).

More than half of the MRSA strains carried SCC*mec *IV or V (Figure 2), which are often seen in community-acquired MRSA (CA-MRSA) [7]. One of the three strains which was previously associated with community-acquisition [23], FIN-4 (ST375:IV, a single locus variant of ST59), was the second most common strain (Table 1). FIN-4 was more likely to be found among isolates obtained from patients and staff members on clinical basis than in screening (167 [21%] of 805 vs. 81 [9%] of 902; p < 0.05) (Table 2) in addition to FIN-16. It has been found in every HD during the study period (range of annual numbers in HDs 7–17). MRSA isolates of ST59 are considered as one of the most divergent MRSA sequence types [9]. ST59 has been reported in a few MSSA isolates from the United States [24], and in a few MRSA isolates in Asian countries [25,26]. ST375, a single locus variant of ST59, has recently been shown to spread as CA-MRSA in Denmark [27]. Unfortunately, no health-care records were available in the present study, and we cannot assess whether acquisition of FIN-4 strain is still associated with community. The two other prevalent SCC*mec *IV expressing strains, FIN-7 (ST8:IV) and FIN-10 (ST45:IV) were more likely to be found in screening specimens of patients and staff members than in their clinical specimens (70 [9%] of 805 vs. 194 [22%] of 902; p < 0.05, and 89 [11%] of 805 vs. 149 [17%] of 902; p < 0.05) (Table 2). FIN-15 (ST8:IV) caused an epidemic in a health-care facility in Eastern Finland in 2000, and since then, the strain has been found occasionally during 2000–2004.

In addition to the presence of SCC*mec *type IV or V, the presence of *lukS-PV *and *lukF-PV*-genes along with epidemiological data are generally used in the definition of CA-MRSA. Of the representative strains, FIN-11 (ST80:IV) and FIN-25 (ST8:IV) were PVL positive (Figure 2). In addition, some strains of FIN-5 (ST30:IV) and FIN-19 (ST1:IV) possessed *lukS*-PV – *lukF*-PV genes encoding PVL. The majority of all MRSA isolates which were previously associated with community-acquisition possessed type IV SCC*mec*, but only a minority (12%) of them were PVL positive [28]. Most of these PVL-positive isolates were of ST80 as reported elsewhere [27,29]. However, although many isolates found in the community harbor these markers, transmission of "CA-MRSA" strains between health-care settings and community may occur [30].

Of FIN-2 strains, FIN-2e (ST241:IIIB) subtype caused an outbreak in Southern Finland in 1999, and only a few strains have been found since then. FIN-2e is a single locus variant of ST239, which is the main ST of FIN-2 strain, and thus it is possible that FIN-2e may have evolved from the FIN-2 by a point mutation. FIN-2 strain includes isolates possessing three different SCC*mec *types (SCC*mec *III, IIIA, and IIIB; Figure 2), indicating that this strain may have received the *mec*A gene several times or SCC*mec *may have been modified. This may be true also with both of FIN-1 and FIN-10 strains which possess two different SCC*mec *types (SCC*mec *IA and IVA, and SCC*mec *IV and V, respectively).

The annual proportion of sporadic strains decreased during the study period, although the total number increased slightly, indicating that new strains appear constantly. Among the patient findings, more than half of the sporadic strains were derived from clinical specimens, 23% from screening specimens based on exposure to MRSA, 20% from screening specimens based on hospital contact abroad, and 2% were other indications (Table 2). The sporadic strains were found most often from specimens taken because of a hospital contact abroad (30%; 19/64). Blanc and co-workers have reported that at least one-third of the sporadic isolates found in Switzerland were due to an ongoing introduction of new isolates from abroad [10]. They have also shown that most of the sporadic Swiss isolates were related to other European epidemic MRSA clones [31]. We cannot be confident of the origin of our sporadic strains because our routine molecular typing scheme is primarily based on the highly discriminatory PFGE, which allows detection of genomic microevolution only. A thorough MLST and eBURST analysis as well as spa typing might have revealed whether our sporadic strains have descended from a few ancestors.

In Finnish acute care hospitals the control of MRSA is based on active culture screening and contact isolation. The increase in the incidence of MRSA remained one of the lowest in Southwestern Finland in comparison with the other regions. In the beginning of the 90's, the first three major MRSA outbreaks in Southwestern Finland and the Helsinki metropolitan area were successfully controlled [32-34]. Later, molecular typing revealed numerous smaller outbreaks [13]. In 2001 the number of MRSA cases rose in elderly persons outside the Helsinki metropolitan area, suggesting an emerging problem in long-term care [20]. Consequently, the national guidelines for control of MRSA were updated to cover also long-term facilities.

Regardless of a strict control policy, the most recent MRSA manifestations in Central and Southern Finland were related to the spread of two internationally recognized MRSA strains, FIN-16 and FIN-21, and these two strains also caused the striking increase in blood and CSF findings. The outbreaks were, however, different in their epidemiology: FIN-16 prevailed in long-term facilities and FIN-21 in acute care hospitals. During the survey period (2001–2003), the proportion of screening specimens increased among the patients and staff members, and almost half of the MRSA isolations were recovered from patients' screening specimens (Table 2), reflecting the active screening policy. The increase in the screening activity may partly be related to the increase in the MRSA cases we detected, especially in cases with FIN-21 [18], but it is unlikely that it had an influence on the worrying trend in blood and CSF findings. In addition, the implementation of control measures may not have been in accordance with the presumptions, which may be due to the lack of resources in some hospital districts or in some settings. Although we had only limited data on clinical infections caused by MRSA, and could not differentiate screening specimens from those taken on clinical basis over the whole study period, we believe that the spread of MRSA has increased, as indicated by each new MRSA case. To guarantee appropriate resources in infection control in all HDs the Ministry of Social Affairs and Health allocated additional funds to MRSA for the years 2005–6. Thus far, this special investment may have paid off since the rising incidence of new MRSA cases seems to have slowed down (data not shown). However, a permanent trend in the near future remains to be seen.

## Conclusion

The predominant MRSA strains change over time, but two internationally spread epidemic strains of MRSA were related to the increase of MRSA incidents detected most recently, and those strains were also the cause of the striking increase of invasive MRSA findings, thus showing the real worsening of the situation in Finland. In addition, the rise in MRSA strains with SCC*mec *types IV or V, possible CA-MRSA, was similar to other European countries [27,29]. The future will show if active control measures will still function in controlling MRSA in this new setting.

## Methods

### Surveillance of MRSA

In Finland (population 5.2 million), the national health care system is organized into 20 geographically and administratively defined hospital districts (HD), with populations ranging from 67.800 to 1.7 million. Fifteen HDs have only secondary and primary care hospitals, and five provide also tertiary care services. Finnish clinical microbiology laboratories notify all MRSA isolations to the National Infectious Disease Register (NIDR) at the National Public Health Institute (KTL). KTL records the date, source of specimen, and the date of birth, sex, and treatment place of the patient. Using this information and a time interval of 36 months, multiple notifications from the same person are combined as one in the database. In case of blood or cerebrospinal fluid (CSF) specimens, the time interval is three months.

### Typing scheme of MRSA

The microbiology laboratories send all MRSA isolates to the National Reference Laboratory (NRL) at KTL for confirmation and typing. The analyses include bacterial identification, antimicrobial susceptibility testing, *mecA *and *nuc *detection by PCR as previously described [20], and genotype analysis by PFGE [19]. PFGE patterns are analyzed by BioNumerics (version 1.0 or 2.0, Applied Maths, Belgium) by using the Dice coefficient to analyze the similarity of the banding patterns, and the unweighted pair group method using arithmetic averages (UPGMA) for cluster analysis. After initial classification by computer assisted analysis, the patterns are further interpreted according to established guidelines [35]. PFGE patterns with 7 or more band differences are considered different types. PFGE patterns with 1–6 band differences are considered as subtypes.

The *S. aureus *PFGE database at KTL is used as the basis for naming the isolates (after naming of the isolates, they are called strains). The database covers all Finnish epidemic MRSA strains, including three strains that have previously been associated with community-acquisition [13,23], and the Harmony collection [19]. If the PFGE profile represents a previously recognized Harmony collection profile, the isolate has been named accordingly. Since 2005, all MRSA strain names have become numeric. Subtypes are additionally labelled with letters. Sporadic strains have been defined as those found only from one person during the study period 1997–2004. Outbreak/epidemic strains of MRSA were isolated from more than one person. For the analysis, only one strain per person was included except with persons showing two or more different strains. Representative isolates, one per PFGE type, were further characterized by MLST [5], and by a multiplex PCR for the determination of the presence of Panton-Valentine leukocidin (PVL) genes (*lukS*-PV – *lukF*-PV) [36], SCC*mec *type I-IV [4], a PCR for the detection of *ccr*-gene and *mec*-gene complex [37], and by PCR for the detection of *ccrC *of type V SCC*mec *[7] as described earlier.

### Control of MRSA

The control measures include screening of patients exposed to MRSA. The minimum of screening sites include nostrils, wounds, and exit sites of devices. Patients are considered exposed if they have shared a room with a MRSA patient or have been hospitalized on a ward where MRSA transmission has occurred from one patient room to another; the latter group covers patients who have been recently hospitalized abroad. Patients with MRSA infection and colonization are nursed in contact isolation (use of apron or gown and gloves) in a single room or in cohorts with designated staff during their hospital stay, the same applies to patient exposed to MRSA until the appearance of MRSA has been ruled out. Staff is screened only if MRSA transmission continues despite ordinary control measures or if the transmission chain remains unclear.

### Survey

During 2001–2003, a more detailed surveillance was performed by collecting demographic and epidemiological data from persons positive for MRSA by sending questionnaires to infection control nurses at the relevant health care institutions. The information collected included 1) whether the MRSA positive person was a patient or a staff member, 2) whether the specimen was taken on clinical or screening basis, 3) whether the patient was screened because of a hospital contact abroad (hospitalized or undergone a surgical procedure outside the Nordic countries within 6 months) or because of an exposure to MRSA, and the staff member because of a hospital contact abroad (worked outside the Nordic countries within 6 months) or because of an epidemic situation, and 4) whether the specimen was taken for some other reason.

### Statistical analysis

For categorical variables, proportions were compared by the chi-square test with Yates correction or Fisher's exact test, as appropriate. The *p*-values of < 0.05 were considered significant.

## Competing interests

The authors declare that they have no competing interests.

## Authors' contributions

A-MK carried the main responsibility of writing the manuscript. She also collected and analyzed the data of the questionnaire survey, analyzed the typing scheme data, and participated in confirmation and typing of MRSA, and in PFGE profile analysis. OL conceived the study and participated in its design and coordination, and participated in writing the manuscript. MK-L participated in the confirmation and typing of MRSA, in PFGE profile analysis, and especially in MLST-typing and analysis. SI participated in the confirmation and typing of MRSA, in PFGE profile analysis, and carried out SCC*mec*-typing. SS participated in the confirmation and typing of MRSA, especially in the analysis of PFGE profiles, was involved in drafting the questionnaire survey, and revision of the manuscript. AV participated in the study design and in writing the manuscript JV-V conceived the study and participated in its design and coordination, and participated in writing the manuscript.

## Pre-publication history

The pre-publication history for this paper can be accessed here:


